# Patients with venous thromboembolism after spontaneous intracerebral hemorrhage: a review

**DOI:** 10.1186/s12959-021-00345-z

**Published:** 2021-11-27

**Authors:** Qiyan Cai, Xin Zhang, Hong Chen

**Affiliations:** 1 Department of Pulmonary and Critical Care Medicine, the First Affiliated Hospital of Chongqing Medical University, No.1 Youyi Road, Yuzhong District, Chongqing, 400016 China; 2 Respiratory Disease Department, Xinqiao Hospital, Chongqing, China

**Keywords:** Intracerebral hemorrhage, Venous thromboembolism, Pulmonary embolism, Deep venous thrombosis, Anticoagulation

## Abstract

**Background:**

Patients with spontaneous intracerebral hemorrhage (ICH) have a higher risk of venous thromboembolism (VTE) and in-hospital VTE is independently associated with poor outcomes for this patient population.

**Methods:**

A comprehensive literature search about patients with VTE after spontaneous ICH was conducted using databases MEDLINE and PubMed. We searched for the following terms and other related terms (in US and UK spelling) to identify relevant studies: intracerebral hemorrhage, ICH, intraparenchymal hemorrhage, IPH, venous thromboembolism, VTE, deep vein thrombosis, DVT, pulmonary embolism, and PE. The search was restricted to human subjects and limited to articles published in English. Abstracts were screened and data from potentially relevant articles was analyzed.

**Results:**

The prophylaxis and treatment of VTE are of vital importance for patients with spontaneous ICH. Prophylaxis measures can be mainly categorized into mechanical prophylaxis and chemoprophylaxis. Treatment strategies include anticoagulation, vena cava filter, systemic thrombolytic therapy, catheter-based thrombus removal, and surgical embolectomy. We briefly summarized the state of knowledge regarding the prophylaxis measures and treatment strategies of VTE after spontaneous ICH in this review, especially on chemoprophylaxis and anticoagulation therapy. Early mechanical prophylaxis, especially with intermittent pneumatic compression, is recommended by recent guidelines for patients with spontaneous ICH. While decision-making on chemoprophylaxis and anticoagulation therapy evokes debate among clinicians, because of the concern that anticoagulants may increase the risk of recurrent ICH and hematoma expansion. Uncertainty still exists regarding optimal anticoagulants, the timing of initiation, and dosage.

**Conclusion:**

Based on current evidence, we deem that initiating chemoprophylaxis with UFH/LMWH within 24–48 h of ICH onset could be safe; anticoagulation therapy should depend on individual clinical condition; the role of NOACs in this patient population could be promising.

## Introduction

Intracerebral hemorrhage (ICH) accounts for approximately 10–30% of all strokes, but is associated with over-proportionally high mortality and enormous health-care costs [1–3]. The most prevalent subtype of ICH is spontaneous ICH, which is principally caused by cerebral small vessel diseases; vascular malformations, tumors, anticoagulants, antiplatelet medication, and vasculitis are among the other reasons [4]. Venous thromboembolism (VTE), including deep venous thrombosis (DVT) and pulmonary embolism (PE), is a common complication in hospitalized patients and is associated with substantial morbidity, mortality, and health-care cost [5]. Patients with ICH have a higher risk of VTE, which can reach 2–4 times that of patients with acute ischemic stroke [6, 7]. In some large database retrospective studies, the incidence of VTE, symptomatic DVT, and clinically evident PE in hospitalized patients with spontaneous ICH has been estimated to be 2–4%, 1–2%, and 0.7–2%, respectively [6–10]. In two prospective studies, the incidence of DVT detected by ultrasonography during hospitalization reached 20–40% [11, 12].

VTE is one of the most common and preventable complications of spontaneous ICH. However, the treatment options of the two are full of contradictions. The focus of VTE treatment is anticoagulation and prevention of recurrent thrombosis, while the treatment of ICH is focused on hemostasis and averting hematoma expansion. For such patients, how should clinicians balance the treatment need of the two conditions and make appropriate treatment strategies is a question worthy of discussion.

In this review, we will briefly summarize the state of knowledge regarding the risk factors, prophylaxis measures, and treatment strategies of VTE after spontaneous ICH.

## Risk factors

### Virchow’s triad

Virchow’s triad describes the three key predisposing factors to VTE: abnormal flow, vessel wall abnormalities (endothelial injury), and coagulation state [13]. In patients with spontaneous ICH, there is venous stasis due to immobility and hemiplegia [14, 15], endothelial injury caused by invasive operations, and hypercoagulability as a result of dehydrating, hemostatic and antifibrinolytic agents [16].

### Other factors

A large number of studies on the risk factors of VTE in patients with spontaneous ICH were conducted; nevertheless, no independent study on this aspect of PE has been performed. Advanced age, immobility, hemiplegia, high D-Dimer and CRP value, high NIHSS score, and previous VTE history as risk factors of VTE in patients with spontaneous ICH have basically reached a consensus [8, 9, 12, 17].

Melmed et al. [18] conducted a study on 414 patients with ICH to clarify the relationship between infection and VTE. They found that respiratory and bloodstream infections were associated with VTE after primary ICH, while urinary and other infections were not.

Whether gender is a relevant risk factor remains controversial. Retrospective studies published by Kim et al. [8] and Ding et al. [9], and the prospective cohort study published by Ogata et al. [12] all pointed out that gender was not a risk factor of VTE. While the prospective cohort studies published by Kawase et al. [11] for the Japanese population and Cheng et al. [17] for the Chinese population indicated that female sex was a risk factor. The discrepancy in the results could be due to differences in design, sample size and population.

## Prophylaxis measures

Patients with spontaneous intracerebral hemorrhage are predisposed to VTE and in-hospital VTE is independently associated with poor outcomes at discharge, 3-month, and 1-year [19]. One study demonstrated that without prophylaxis, up to 75% of patients with residual hemiplegia following ICH developed DVT, and PE-related deaths occurred in approximately 5% of patients with ICH [20]. As a consequence, the prophylaxis of VTE is of vital importance. Prophylaxis measures can be categorized into active limb movement, mechanical prophylaxis, and chemoprophylaxis. Active limb movement, such as ankle pump exercise (APE) which rhythmically contracts and relaxes the calf muscles through the ankle joint movement, and squeezes the venous plexus to promote the venous blood return in lower limbs, is efficient and cost-effective in eliminating venous stasis to contribute to the prophylaxis of DVT [21–23]. We mainly focus on mechanical and chemical prophylaxis in this review.

### Mechanical prophylaxis

For some hospitalized medical patients at high risk of thrombosis (e.g. patients with hemorrhagic stroke [7], lower extremity fractures [24], nephrotic syndrome [25], etc.) who are bleeding or at high risk of major bleeding, the guideline from American College of Chest Physicians (ACCP) has suggested the optimal use of mechanical prophylaxis with graduated compression stockings (GCS) or intermittent pneumatic compression (IPC), rather than no mechanical prophylaxis [26].

#### GCS and IPC

For the prophylaxis of VTE, guidelines published in recent years recommended the use of IPC and/or GCS (Table 1). The Clots in Legs Or sTockings after Stroke (CLOTS) trial 1, a multicenter and randomized controlled trial in stroke patients who were enrolled between 2001 and 2008 (*n* = 2518, 232 with ICH), demonstrated that GCS alone was ineffective in preventing DVT (10.0% vs. 10.5%, OR 0.98, 95%CI 0.76–1.27) and caused more adverse effects, including skin breaks, ulcers, blisters, and necrosis of lower extremities (5.0% vs. 1.0%, OR 4.18, 95%CI 2.40–7.27) [32]. The CLOTS trial 3, which was also a multicenter and randomized controlled trial conducted between 2008 to 2012 in patients who were immobile after stroke (*n* = 2876, 376 with ICH) to assess the effectiveness of IPC in preventing DVT, addressed that the routine application of IPC in patients with ICH was associated with a significantly decreased risk of DVT (6.7% vs. 17.0%, OR 0.36, 95%CI 0.17–0.75) [33]. Therefore, recent guidelines have tended to recommend IPC alone.

**Table 1:** Recommendations for GCS and IPC in recent guidelines for patients with spontaneous ICH

Years	Organization	GCS	IPC	Timing	Grade
2012	ACCP [26]	√	√	Not mentioned	Weak recommendation, low-quality evidence
2014	ESO [27]	×	√	Not mentioned	Strong recommendation, moderate-quality evidence
2015	AHA/ASA [28]	×	√	At admission	Strong recommendation, high-quality evidence
2016	NCS [29]	√	√	At admission	Strong recommendation, high-quality evidence
2018	NICE [30]	×	√	Within 3 days	Not mentioned
2020	HSFC [31]	×	√	At admission	High-quality evidence

*ACCP* the American College of Chest Physicians; *ESO* European Stroke Organization; *AHS/ASA* the American Heart Association/American Stroke Association; *NC*S the Neurocritical Care Society; *NICE* National Institute for Health and Care Excellence; *HSFC* Heart and Stroke Foundation of Canada

√ recommended, × not recommended

#### Timing of initiation

Regarding the timing of intervention for mechanical prophylaxis in patients with spontaneous ICH, the recommendations of the current guidelines (Table 1) have tended to converge: start at the time of hospital admission and use as early as possible. In 2018, the guideline from NICE added recommendations that mechanical prophylaxis should be initiated within 3 days of ICH [30].

### Chemoprophylaxis

The use of chemoprophylaxis with unfractionated heparin (UFH), low molecular weight heparin (LMWH), and direct oral anticoagulants (e.g. warfarin, rivaroxaban, apixaban, dabigatran, etc.) in patients with spontaneous ICH is an area of ongoing debate. Determining appropriate chemoprophylaxis is challenging in the presence of ICH. The concern of hematoma expansion and recurrent ICH limits the use of chemoprophylaxis in clinical practice. A large database retrospective study including 32,690 patients with spontaneous ICH from 2006 to 2010 demonstrated that 5395 patients (16.5%) received chemoprophylaxis [34]. Another multicenter observational cohort study demonstrated that among 74,283 patients with ICH, only 5929 received chemoprophylaxis while 66,444 received mechanical prophylaxis (7.9% vs. 89.4%) [35].

#### Timing of initiation

To date, the optimal timing for the initiation of chemoprophylaxis is still uncertain. A large database retrospective study demonstrated 44.8% (2416/5395) of patients with spontaneous ICH received prophylactic anticoagulation with UFH, enoxaparin, or dalteparin by day 2 of onset [34]. Hematoma expansion usually occurs in the early phase of ICH. Results from imaging studies have demonstrated that 70% of patients with spontaneous ICH have hematoma expansion within 24 h of onset and expansion after 24 h seems extremely rare [36, 37]. In contrast, patients with ICH develop DVT as early as the second day without prophylaxis, with the peak incidence occurring between days 2 and 7 [38]. In addition, anticoagulation is associated with recurrent ICH in patients with active bleeding, while no such association has been found in patients who have documentation of hematoma stabilization on neuroimaging [39]. Therefore, we speculate that initiating chemoprophylaxis within 24–48 h of onset, under the circumstance of no signs of hematoma expansion or active bleeding on neuroimaging, could be safe and effective. A number of studies (Table 2) demonstrated that initiating chemoprophylaxis with UFH/LMWH within 24–48 h was safe for being not associated with hematoma expansion or recurrent ICH, but differed in the efficiency. Initiating chemoprophylaxis within 24–48 h seems not to be more effective in reducing the risk of VTE than other timing. Recommendations in relevant guidelines vary as well (Table 3). Hence, more high-quality studies are needed on the timing of initiating chemoprophylaxis among patients with spontaneous ICH.

**Table 2:** Studies about chemoprophylaxis in patients with spontaneous ICH

First author (year)	Design	N of pts	Timing	Prophylaxis	Risk of VTE	Risk of HE /recurrent ICH
Boeer [40] (1991)	Prospective	68	2 days vs. 4 days vs. 10 days	UFH 5000 U q8h	Reduction in number of PE in 2 days group (1 vs. 5 vs. 9)	No significant difference
Wasay [41] (2008)	Prospective	458	1–6 days	UFH 2500-5000 U bid+GCS vs. GCS alone	No significant difference	No significant difference
Orken [42] (2009)	Randomized trial	75	48 h	Enoxaparin 4000 IU qd vs. GCS	No significant difference	No significant difference
Kiphuth [43] (2009)	Retrospective	97	< 36 h	Enoxaparin 4000 IU qd or dalteparin 2500 IU qd	Not mentioned	0 at day 2
Faust [44] (2017)	Retrospective	400	<48 h vs. ≥48 h	Not mentioned	0.7% vs. 3.1% (*P* = 0.17)	5.6% vs. 5.0% (*P* = 0.80)
Ianosi [45] (2019)	Retrospective	134	<48 h vs. ≥48 h	Enoxaparin 2000 IU/4000 IU qd	Not mentioned	No significant difference
Kananeh [46] (2021)	Retrospective	163	<24 h vs. ≥24 h	UFH 5000 U q8h	Not mentioned	3.4% vs. 6.7% (*P* = 0.49)
Qian [47] (2021)	Randomized trial	139	24 h vs. 72 h	Enoxaparin 2000 IU bid	No significant difference	No significant difference

*N of pts* number of patients; *UFH* unfractionated heparin*; GCS* graduated compression stockings*; HE* hematoma expansion

**Table 3:** Recommendations for chemoprophylaxis in recent guidelines for patients with spontaneous ICH

Years	Organization	LMWH	UFH	Timing	Grade
2012	ACCP [26]	√	√	2–4 days	Weak recommendation, low-quality evidence
2014	ESO [27]	Not mentioned	Not mentioned	Not mentioned	Weak recommendation, low-quality evidence
2015	AHA/ASA [28]	√	√	Not mentioned	Weak recommendation, moderate-quality evidence
2016	NCS [29]	√	√	Within 48 h	Weak recommendation, low-quality evidence
2020	HSFC [31]	√	Not mentioned	After 48 h	Moderate-quality evidence

*ACCP* the American College of Chest Physicians; *ESO* European Stroke Organization; *AHS/ASA* the American Heart Association/American Stroke Association; *NC*S the Neurocritical Care Society; *HSFC* Heart and Stroke Foundation of Canada; *UFH* unfractionated heparin; *LMWH* low molecular weight heparin

√ recommended, × not recommended

#### Anticoagulants

Anticoagulants mostly used in relevant studies are UFH or LMWH. A large database retrospective study on patients with spontaneous ICH from 2006 to 2010 (*n* = 32,690) showed that the most commonly used agents for VTE prophylaxis were heparin (71.1%), enoxaparin (27.5%), and dalteparin(1.4%) [34]. The efficacy and safety of most direct oral anticoagulants (DOACs) in preventing VTE in patients with spontaneous ICH have not been evaluated because they have been excluded from randomized trials on VTE chemoprophylaxis with DOACs. The majority of studies **(**Table 2**)** have demonstrated that UFH or LMWH is not associated with recurrent ICH, hematoma expansion, and increased mortality. A meta-analysis, including 9 studies and 4055 patients with spontaneous ICH, also showed that chemoprophylaxis with UFH/LMWH was not associated with a significant increase in hematoma expansion (6.6% vs. 3.2%, *P* = 0.14) or mortality (12.0% vs. 11.8%, *P* = 0.29) in comparison with non-heparin prophylaxis [48]. However, the efficacy of VTE chemoprophylaxis has not reached a consensus (Table 2).

#### Dosage

The appropriate anticoagulant dosage is still inconclusive. For VTE prophylaxis with UFH, the suggested dosage is 5000 units subcutaneously two or three times daily. No consensus has been established regarding the optimal frequency of dosing (two vs. three times daily). While with LMWH, Tetri et al. retrospectively evaluated the safety and efficacy of enoxaparin in VTE prophylaxis among 407 patients with spontaneous ICH and demonstrated that the appropriate dosage could be 2000 or 4000 IU once daily [49]. Wu et al. [50] also pointed out that subcutaneous administration of 4000 IU of enoxaparin once daily can prevent DVT in patients with ICH without hematoma expansion or recurrent ICH.

In conclusion, controversies exist regarding the efficacy, optimal anticoagulants, timing, and dosage of VTE chemoprophylaxis for patients with spontaneous ICH. Recommendations vary among relevant guidelines (Table 3). We expect more high-quality studies to provide direction for clinical decision-making.

## Treatment strategies

The treatment strategies for VTE include anticoagulation, vena cava filter, systemic thrombolytic therapy, catheter-based thrombus removal, and surgical embolectomy. Controversies and uncertainties remain in the treatment of VTE after spontaneous ICH, especially in the acute phase of ICH (usually within 2 weeks of onset) which has a high risk of hematoma expansion and recurrence [51]. We briefly summarize the evidence of VTE treatment after acute spontaneous ICH as follows.

### Anticoagulation

Anticoagulation therapy is the cornerstone of VTE treatment. However, active bleeding is considered a contraindication to anticoagulation therapy. Decision-making on anticoagulation therapy after spontaneous ICH evokes debate among clinicians and the inadequacies in the evidence, for the reason that such patients have been excluded from randomized trials of anticoagulation therapy for VTE, makes the decision-making challenging. The three questions that need to be answered are as follows: first, whether anticoagulation therapy should be applied on such patients; second, when is the appropriate timing to initiate anticoagulation; and third, which anticoagulant is the optimal choice.

#### Anticoagulation or not

Limited data are available on the pros and cons of anticoagulation therapy for VTE following spontaneous ICH. Whether anticoagulation should be used depends on the individual risk-benefit ratio of anticoagulation therapy, that is, the risk of recurrent ICH and hematoma expansion versus the risk of VTE progression.

No reliable methods have been established to predict the risk of recurrent ICH and hematoma expansion. The pathophysiological mechanism of hematoma expansion remains unclear. A number of studies have demonstrated that previous use of anticoagulants or antiplatelet agents, advanced age, systolic hypertension, hyperglycemia, high NIHSS score or Glasgow Coma Scale score are related clinical risk factors for hematoma expansion [52–61]; CTA spot sign, blend sign, black hole sign, island sign, and iodine sign are the radiological risk factors [62–68]. The annualized rate of recurrent spontaneous ICH is approximately 2% [1, 69]. Spontaneous ICH principally results from small vessel diseases that are mainly composed of hypertensive arteriopathy and cerebral amyloid angiopathy (CAA). Hypertension has been identified as one of the most crucial and modifiable risk factors for recurrent ICH. Lowering blood pressure is associated with a reduced recurrence of ICH [70–72]. Amyloid angiopathy has a predilection for the cortical arteries, hence CAA is a major contributor of spontaneous lobar ICH, which has a significantly higher risk for recurrence compared to deep ICH [73–75]. In addition, a number of studies have demonstrated that older age, cerebral microbleeds (CMB) and cortical superficial siderosis (cSS) on MRI, and Apolipoprotein E (APOE) genotype (ε2 or ε4) are associated with a higher recurrence risk [76–78]. In conclusion, anticoagulation therapy should be more careful in patients with the risk factors mentioned above. Besides, the modifiable risk factors, such as hypertension and hyperglycemia, should be minimized if anticoagulants are to be used in this patient population.

Patients with spontaneous ICH have been excluded from prospective studies and randomized trials on anticoagulation therapy for VTE. The available literature is almost exclusively case reports. Ajmeri et al. [79] presented a case of a 68-year-old male who suffered from recent ICH and recurrent PE. The anticoagulation therapy was initiated with UFH after a CT scan was done to eliminate active bleeding, and then altered to enoxaparin. The patient was discharged from the hospital in a stable condition without neurological deficits. Similar cases were also reported by Becattini et al. [80] and Lee et al. [81]

A Danish large-database retrospective study (*n* = 2978) which was focusing on patients with spontaneous ICH, demonstrated that oral anticoagulant (OAC) resumption was associated with decreased risk of thrombotic events and not increasing the risk of recurrent ICH [82]. Current studies on anticoagulation therapy are mostly focused on the OAC resumption after anticoagulation-related ICH. In addition to VTE, the indications for anticoagulation resumption include atrial fibrillation, mechanical valves, myocardial infarction, etc. The majority of studies have tended to reach a consensus that anticoagulation resumption results in a clinical benefit in terms of thromboembolic event reduction without increasing the risk of recurrent ICH or hematoma expansion (Table 4). These results were confirmed by a meta-analysis (8 studies, 5306 patients with ICH) that demonstrated restarting OAC decreased the risk of thrombotic events (6.7% vs. 17.6%, RR 0.34, 95%CI 0.25–0.45) without significantly increased risk of recurrent ICH (8.7% vs. 7.8%, RR 1.01, 95%CI 0.58–1.77) [87]. What is noteworthy is that only the patients with smaller hemorrhage volumes and mild functional changes have been eligible for anticoagulation resumption in many studies, consequently decreasing the risk of recurrent ICH.

**Table 4:** Studies on anticoagulation resumption after ICH

First author (year)	Design	ICH type	N of pts	Indications	Timing of resumption	Anticoagulants	Comparator	Risk of TE	Risk of recurrent ICH
Majeed [83] (2010)	Retrospective	Anticoagulation-related	234	AF, MV, VTE	Median 5.6 weeks	Warfarin	Without AC	Not mentioned	8 vs. 10 (HR 5.6, 95%CI 1.8–17.2)
Yung [84] (2012)	Retrospective	Anticoagulation-related	284	AF, MV, VTE	Within a month	Warfarin	Without AC	Not mentioned	15.4% vs. 15.0% (*P* = 0.94)
Kuramatsu [85] (2015)	Retrospective	Anticoagulation-related	719	AF, MV, VTE	Median 31 days	OAC	Without AC	5.2% vs. 15.0% (*P* < 0.001)	8.1% vs. 6.6% (*P* = 0.48)
Witt [86] (2015)	Retrospective	Anticoagulation-related	160	AF, MI, MV, IS, VTE	Median 14 days	Warfarin	Without AC	3.7% vs. 12.3% (*P* = 0.092)	7.6% vs.3.7% (*P* = 0.497)
Ottosen [82] (2016)	Retrospective	Spontaneous	2978	AF, IS, MI, MV, PAD, VTE	Not mentioned	OAC	Without AC	Lower (HR 0.58, 95%CI 0.35–0.97)	Not increased (HR 0.90, 95%CI 0.44–1.82)

*N of pts* number of patients; *AF* atrial fibrillation; *MV* mechanical valves; *MI* myocardial infarction; *IS* ischemic stroke; *PAD* peripheral vascular disease; *OAC* oral anticoagulants; *AC* anticoagulants; *TE* thrombotic events; *HR* hazard ratio

Nevertheless, some opposite results exist. A retrospective study with 79 patients who had brain tumors and following anticoagulation-related ICH showed that anticoagulation resumption with LMWH or DOACs was associated with a significantly lower risk of recurrent VTE (8.1% vs. 35.3%, *P* = 0.003) but a higher risk of recurrent ICH (6.1% vs. 4.2%), especially in patients with primary brain tumors and major ICH [88]. Different study designs and research objects may explain the opposite results. Several retrospective studies and meta-analysis studies have shown that anticoagulation is associated with an increased risk of ICH in patients with primary brain tumors, however, the association seems not to be found in patients with metastatic brain tumors [89–91]. Hence, anticoagulation resumption in patients with ICH and primary brain tumors must be cautious.

#### Timing of initiation

No consensus exists on optimal timing for initiation of anticoagulation therapy following spontaneous ICH. The timing ranged from 2.5 days to 18 weeks in retrospective studies on anticoagulation resumption after ICH [92]. No randomized trial data are available to guide the decision. Hence, the timing of initiating anticoagulation therapy should depend on the individual clinical conditions.

#### Anticoagulant choice

No comparative studies on different anticoagulants in patients with VTE after spontaneous ICH have been published. Existing studies mostly focus on the safety of anticoagulants and ICH is one of the crucial indicators. The relevant studies almost exclusively referred to vitamin-K antagonists used for anticoagulation resumption after ICH. However, the role of novel oral anticoagulants (NOACs), including dabigatran (inhibitor of factor IIa), rivaroxaban, apixaban, and edoxaban (inhibitors of factor Xa), may be more promising than vitamin-K antagonists for the reasons as follows: (1) NOACs are more convenient to use for lack of monitoring requirements and less interaction with food and other drugs; (2) a number of large-scale retrospective studies and randomized trials have proved that NOACs are associated with a reduction in the risk of ICH compared with vitamin K antagonists **(**Table 5**)**; (3) NOACs-related ICH is less severe than warfarin-related ICH, with smaller hematoma volume, lower rate of hematoma expansion, favorable functional and vital outcomes, and lower mortality [98–100]; (4) the availability of antidotes (idarucizumab and andexanet) that allow an immediate and complete reversal of the anticoagulant effect of NOACs [101–103]. We expect high-quality and large-scale studies on patients with spontaneous ICH to provide support.

**Table 5:** Studies about NOACs vs. warfarin in patients with VTE

First author (year)	Design	N of pts	Anticoagulant	Comparator	Recurrent VTE and VTE-related death	Risk of ICH
Schulman [93] (2009)	Randomized trial	2564	Dabigatran	Warfarin	2.4% vs. 2.1%, HR 1.10 (0.65–1.84)	0 vs. 3
Buller [94] (2012)	Randomized trial	4832	Rivaroxaban	Enoxaparin, followed by warfarin	2.1% vs. 1.8%, HR 1.12 (0.75–1.68)	3 vs. 12
Agnelli [95] (2013)	Randomized trial	5395	Apixaban	Enoxaparin, followed by warfarin	2.3% vs. 2.7%, HR 0.84 (0.60–1.18)	3 vs. 6
Buller [96] (2013)	Randomized trial	4921	Edoxaban	Warfarin	3.2% vs. 3.5%, HR 0.89 (0.70–1.13)	0 vs. 6
Lamsam [97] (2018)	Retrospective	218,620	NOACs	Warfarin	Not mentioned	1/1000 vs. 3.3/1000 (*P* < 0.0001)

*N of pts* number of patients; *NOACs* novel oral anticoagulants; *HR* hazard ratio

### Inferior vena cava filter

The efficacy of inferior vena cava (IVC) filter in preventing formation or aggravation of PE caused by shedding of deep vein thrombosis of lower extremities has been confirmed in general patients. However, none of the studies have focused solely on patients with spontaneous ICH.

A retrospective study included 371 stroke patients (including 105 patients with hemorrhagic stroke) who received IVC filters and demonstrated that IVC filters were effective in preventing life-threatening PE; however, the filter-related complications, including filter migration, filter fracture, and filter thrombosis, were also worthy of attention [104]. Meritxell et al. [105] used the data of the RIETE registry to figure out the association between the use of IVC filters and the outcome of patients presenting with major bleeding during anticoagulation for VTE. Among 1065 patients (including 124 patients with intracranial hemorrhage) in this study, 11% received IVC filters; the result suggested that patients receiving IVC filters had a lower risk for all-cause death (HR 0.55, 95%CI 0.23–1.40) and a similar risk for PE recurrence (HR 1.57, 95%CI 0.38–6.36). However, the study by Kare et al. [106] on 1068 patients with acute brain injury (the proportion of ICH was not mentioned) who received IVC filters demonstrated that IVC filters were ineffective in preventing PE (HR 3.19, 95%CI 1.3–3.3) and reducing mortality (HR 1.0, 95%CI 0.8–1.3). Besides, once IVC filters were placed, few were removed. Jan et al. [107] also supported this result. Among 204 retrievable IVC filters inserted in neurosurgical patients in their study, only 19% were retrieved and 55% converted to permanent devices. We deem that the difference in results is partly due to the different patient groups included in each study. Therefore, whether the IVC filters are beneficial to patients with VTE after spontaneous ICH still needs targeted studies.

### Other treatments

The application of systemic thrombolytic therapy, catheter-based thrombus removal, and surgical embolectomy in patients with VTE after spontaneous ICH is only seen in a few case reports and still needs further exploration.

Current guidelines have recommended the immediate administration of thrombolytic therapy in patients with massive PE and without contraindications [26]. Thrombolytic therapy is said to be contraindicated in the presence of ICH. However, some case reports revealed that thrombolysis could be safe and effective in patients with the two conditions. Wendy et al. [108] presented a case of a 60-year-old woman with massive PE (led to cardiac arrest with pulseless electrical activity) and a recent hemorrhagic cerebrovascular accident who was administered tissue plasminogen activator (t-PA). No hematoma expansion occurred, and the patient was eventually discharged from the hospital. In another report, systemic thrombolysis with recombinant tissue plasminogen activator (rt-PA) for massive PE was used with success in a 53-year-old male with acute spontaneous ICH [109]. Therefore, in patients who are at high risk of death from massive pulmonary embolism, contraindications to thrombolysis should be weighed against the potential benefit.

The ACCP guideline recommends catheter-based thrombus removal or surgical embolectomy in patients with acute PE who have hypotension and contraindications to thrombolysis if appropriate expertise and resources are available [26]. No studies or case reports have been reported in the application of catheter-based thrombus removal in patients with VTE after spontaneous ICH as yet. For surgical methods, Endo et al. [110] reported a 69-year-old presented with acute PE 11 days after ICH and underwent surgical embolectomy with cardiopulmonary bypass. No recurrence of ICH was observed in this patient.

### Patients with preexisting VTE and ICH

Although this review mainly focuses on the prophylaxis and treatment of VTE after spontaneous ICH, another crucial and overlapping clinical scenario that happens in patients with preexisting VTE and following ICH is also noteworthy. Anticoagulation-related ICH is more common in this patient population due to the need for long-term anticoagulation therapy of VTE. Anticoagulation-related ICH is more severe and associated with a higher risk of hematoma expansion and higher mortality, compared to other spontaneous ICH [56, 85, 111]. Managements of anticoagulation-related ICH include halting anticoagulation and anticoagulants reversal, which has protamine sulfate, vitamin K, fresh frozen plasma (FFP), prothrombin complex concentrate (PCC), rFVIIa, idarucizumab, and andexanet as options for different anticoagulants [112]. In addition, anticoagulation resumption should also be considered in patients with preexisting VTE. Anticoagulation should be restarted when the risk of VTE progression outweighs recurrent ICH or hematoma expansion. However, as the description in Section 4.1, the optimal timing and anticoagulants remain unclear.

## Conclusion

1. Early mechanical prophylaxis with IPC and/or GCS is recommended, having a preference for IPC. Controversies exist regarding the effectiveness, optimal anticoagulants, timing, and dosage of VTE chemoprophylaxis. Initiating chemoprophylaxis with UFH/LMWH within 24–48 h of spontaneous ICH onset could be safe.
2. Given the limited evidence and the observational nature of the studies regarding anticoagulation therapy, uncertainty exists regarding anticoagulation, timing, and anticoagulants. Anticoagulation treatment should depend on individual clinical condition. The role of NOACs in this patient population could be promising.
3. More targeted and high-quality studies on vena cava filter, systemic thrombolytic therapy, catheter-based thrombus removal, and surgical embolectomy are needed.

## Data Availability

Not applicable.

## Abbreviations

ACCP: the American College of Chest Physicians
AF: Atrial fibrillation
AHS/ASA: the American Heart Association/American Stroke Association
DOACs: Direct oral anticoagulants
DVT: Deep venous thrombosis
ESO: European Stroke Organization
GCS: Graduated compression stockings
HE: Hematoma expansion
HR: Hazard ratio
HSFC: Heart and Stroke Foundation of Canada
ICH: Intracerebral hemorrhage
IPC: Intermittent pneumatic compression
IVC: Inferior vena cava
LMWH: Low molecular weight heparin
MI: Myocardial infarction
MV: Mechanical valves
NCS: The Neurocritical Care Society
NICE: National Institute for Health and Care Excellence
OAC: Oral anticoagulants
PAD: Peripheral vascular disease
PE: Pulmonary embolism
UFH: Unfractionated heparin
VTE: Venous thromboembolism
